# Online Training and Self-assessment in the Histopathologic Classification of Endocervical Adenocarcinoma and Diagnosis of Pattern of Invasion: Evaluation of Participant Performance

**DOI:** 10.1097/PGP.0000000000000757

**Published:** 2021-02-09

**Authors:** Kay J. Park, Isabel A. Cabrero, Oluwole Fadare, Lynn Hoang, Takako Kiyokawa, Esther Oliva, Carlos Parra-Herran, Joseph T. Rabban, Andres Roma, Naveena Singh, Robert Soslow, Simona Stolnicu, Jutta Huvila, Samuel Leung, C. Blake Gilks

**Affiliations:** Department of Pathology, Memorial Sloan Kettering Cancer Center, New York, New York (K.J.P., R.S.); Hospital de Oncologia, CMN, SXXI, Instituto Mexicano del Seguro Social, Mexico City, Mexico (I.A.C.); Department of Pathology, University of California San Diego, San Diego (O.F., A.R.); Department of Pathology, University of California San Francisco, San Francisco (J.T.R.), California; Department of Pathology and Laboratory Medicine, Vancouver General Hospital and University of British Columbia, Vancouver, British Columbia, Canada (L.H., S.L., C.B.G.); Department of Pathology, The Jikei University School of Medicine, Tokyo, Japan (T.K.); Department of Pathology, Massachusetts General Hospital (E.O.); Department of Pathology, Brigham and Women’s Hospital and Harvard Medical School (C.P.-H.), Boston, Massachusetts; Department of Cellular Pathology, Barts Health NHS Trust, London, UK (N.S.); Department of Pathology, University of Medicine, Pharmacy, Sciences and Technology of Targu Mures, Romania (S.S.); Department of Pathology, University of Turku, Turku, Finland (J.H.)

**Keywords:** Endocervical adenocarcinoma, IECC classification, Silva pattern, WHO classification

## Abstract

Histopathologic classification of endocervical adenocarcinomas (EAC) has recently changed, with the new system based on human papillomavirus (HPV)-related morphologic features being incorporated into the 5th edition of the WHO Blue Book (*Classification of Tumours of the Female Genital Tract*). There has also been the introduction of a pattern-based classification system to assess invasion in HPV-associated (HPVA) endocervical adenocarcinomas that stratifies tumors into 3 groups with different prognoses. To facilitate the introduction of these changes into routine clinical practice, websites with training sets and test sets of scanned whole slide images were designed to improve diagnostic performance in histotype classification of endocervical adenocarcinoma based on the International Endocervical Adenocarcinoma Criteria and Classification (IECC) and assessment of Silva pattern of invasion in HPVA endocervical adenocarcinomas. We report on the diagnostic results of those who have participated thus far in these educational websites. Our goal was to identify areas where diagnostic performance was suboptimal and future educational efforts could be directed. There was very good ability to distinguish HPVA from HPV-independent adenocarcinomas within the WHO/IECC classification, with some challenges in the diagnosis of HPV-independent subtypes, especially mesonephric carcinoma. Diagnosis of HPVA subtypes was not consistent. For the Silva classification, the main challenge was related to distinction between pattern A and pattern B, with a tendency for participants to overdiagnose pattern B invasion. These observations can serve as the basis for more targeted efforts to improve diagnostic performance.

There has been a significant change in the basis for the classification of endocervical adenocarcinomas in the fifth edition of the WHO Blue Book (*Classification of Tumours of the Female Genital Tract*) 1, with adoption of the International Endocervical Adenocarcinoma Criteria and Classification (IECC) system 2–6. This classification is based on etiology [human papillomavirus (HPV) associated (HPVA) or not], with further subclassifcation of HPV-independent (HPVI), also referred to as non-HPVA or NHPVA adenocarcinomas into a number of subtypes that have characteristic histopathologic, immunophenotypic, and molecular abnormalities. The HPVA adenocarcinomas show a range of architectural and cytologic features 1,7. In contrast, in the fourth edition of the WHO, classification of endocervical adenocarcinomas was based purely on morphology, and one of the major categories, mucinous carcinoma, included both HPVA and HPVI tumors. On the basis of data published since the fourth edition it is now appreciated that the HPVA and HPVI differ not only in etiology, but also in prognosis, thus the change 2–6. A system to assess the pattern of invasion of endocervical adenocarcinomas has also been developed and validated 7–14; it is applicable to HPVA but not HPVI carcinomas 13. Silva and colleagues identified 3 patterns of invasion, A, B, and C, each associated with significantly different prognoses.

To facilitate the translation of these new classification systems into clinical practice the International Society of Gynecological Pathologists is undertaking a multipronged project, including a large international collaborative study of endocervical adenocarcinoma outcomes. In support of this project 2 websites were created and introduced to the society membership at the 2020 USCAP ISGyP Companion Society Meeting. The goal was to provide pathologists with training and self-assessment modules in the use of these 2 new systems, through written guidance on their use and whole slide scanned images of cases selected by experts, to allow for more rapid adoption and accurate use of the new classification systems. In this manuscript we report on the initial results of participants who have used these websites. Although the primary goal in creating the websites was to provide an educational tool to promote rapid and accurate adoption of the new classification systems, a secondary goal was to identify areas for potential diagnostic improvement, by monitoring aspects of diagnosis that were most challenging for participants.

## MATERIALS AND METHODS

Two websites were created, both consisting of whole slide scanned images of endocervical adenocarcinoma. Scanned slides were provided by one pathologist (K.J.P.) from the archives of MSKCC. A single hematoxylin & eosin–stained slide was selected for each case. No immunohistochemical stained slides were included on the website nor was any information provided regarding the results of any immunohistochemical testing that may have been performed for the original clinical diagnosis. No information about the patient age, HPV status, clinical or pathologic stage or outcome was provided. The digital slides were accessible by browser-embedded viewing software that enabled full navigation of the slide by panning and zoom up to 40× magnification. Two different sets of cases were used to create the 2 websites; one for training/assessment in the use of histotype diagnosis according to the WHO 2020/IECC, and another for training/assessment in the diagnosis of Silva pattern of invasion. The design of the websites was based on websites previously created as an aid to learning ovarian carcinoma histotype diagnosis (http://www.gpecimage.ubc.ca/aperio/images/transcanadian/) 15, assignment of Chemotherapy Response Scores in high-grade serous carcinoma treated with neoadjuvant chemotherapy (http://www.gpecimage.ubc.ca/aperio/images/crs) 16, and assessment of p53 immunostaining patterns in endometrial carcinoma (http://www.gpec.ubc.ca/p53) 17.

Both websites were first tested by gynecologic pathologists with experience using the IECC system and the Silva classification system, in order to confirm the reference diagnosis, and to identify any slides that did not show diagnostic features. ISGyP members who came forward participate in the international EAC outcomes study were sent the link to the 2 educational websites on December 21, 2019. At the 2020 USCAP ISGyP Companion Society meeting the 2 websites were unveiled to the membership and an invitation was issued through the Society for interested parties to try them; also an invitation to use the websites was sent to all members of the Society when they were invited to share data on their endocervical adenocarcinoma cases, a study that has not yet been completed. All participation was anonymous, and the results were tabulated through the website. Results up to August 13, 2020 are presented.

### Endocervical Adenocarcinoma: Histopathologic Classification

**FIG. 1:**
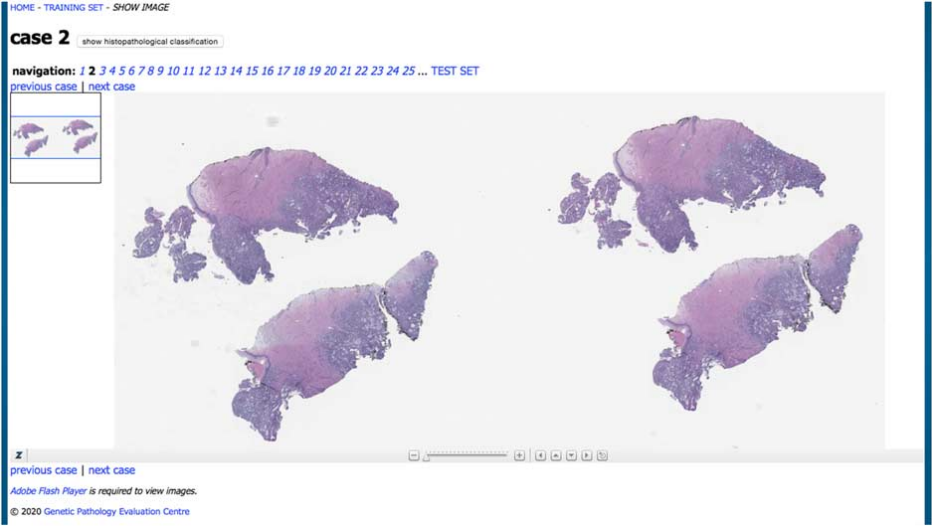
A screenshot of the website for histopathologic classification of endocervical adenocarcinomas according to the WHO 2020/IECC system. For this case from the training set the reference diagnosis can be accessed while viewing the slide, whereas for the test set the diagnoses are only made available when all slides in the test set have been reviewed and diagnoses entered. Genetic Pathology Evaluation Centre, Vancouver, BC. All permission requests for this image should be made to the copyright holder.

This website included a training set of 25 scanned slides and 28 test slides; for the former the diagnosis could be seen by participants at the time they viewed the slide (Fig. 1), whereas for the latter 28 slides no diagnosis was provided; instead participants selected a diagnosis from a drop-down menu and only when the test set was completed would the participant receive their tabulated results, indicating which cases they diagnosed correctly or incorrectly. The training set includes 13 HPVA and 12 HPVI endocervical adenocarcinomas. Upon going to the website, http://www.gpec.ubc.ca/eac2, and before viewing the scanned slides, the participants received written guidelines on how to classify tumors according to the WHO 2020/IECC criteria (written by R.S. and E.O.) (Table 1). For analysis, the participants’ agreement with the following diagnostic categories was compared: (1) HPVA versus HPVI including any subtype (2) subtype of HPVA, and (3) subtype of HPVI.

**TABLE 1:** Histopathologic classification of endocervical adenocarcinoma

The purpose of this website is to provide training in performing histopathologic classification of endocervical adenocarcinoma. This is done by diagnosis of one of the histotypes of endocervical adenocarcinoma, using the criteria below: HPV-associated endocervical adenocarcinomas (HPVA) ∼85% of all endocervical adenocarcinomas in an international series Easily identified apical mitotic figures and karyorrhexis (apoptotic bodies) at 40× magnification Confirmation of HPV status is optional; it can be performed with HR-HPV mRNA-ISH, HPV-PCR, or p16. mRNA-ISH test has superior sensitivity and specificity. p16 immunostaining is a good surrogate marker provided one is cognizant of its imperfect specificity Morphologic variants include: Usual Glandular, cystic, cribriforming, papillary, microglandular, solid patterns Extravasated mucin may be seen Pseudostratified, enlarged, elongated, and hyperchromatic nuclei Conspicuous apical mitoses and apoptotic bodies at scanning magnification Apical amphophilic to eosinophilic cytoplasm with <50% of tumor cells containing intracytoplasmic mucin (mucin depleted) Papillary (including villoglandular) Exophytic growth with long, slender papillae One or several layers of tall mucin-poor endocervical or intestinal-type epithelium Mild cytologic atypia and variable mitotic activity Fibrovascular cores contain spindle cells and frequent acute and chronic inflammatory cells Mucinous Not otherwise specified: ≥50% of tumor cells with intracytoplasmic mucin in a background of usual endocervical-type adenocarcinoma No specific features of gastric, intestinal, or signet ring cell morphology Intestinal (with goblet cells, sometimes with neuroendocrine differentiation): ≥50% of cells with goblet morphology in a background of usual endocervical-type adenocarcinoma It may have argentaffin and Paneth cells Signet ring cell: ≥50% of cells with signet ring cell morphology in a background of usual endocervical-type adenocarcinoma Diffuse, trabecular, glandular, and cord-like growths Invasive stratified mucinous carcinoma: Invasive nests or trabeculae of pseudostratified pale columnar cells with peripheral palisading and variable intracytoplasmic mucin (closely resembling morphologically SMILE-in situ counterpart) Intracytoplasmic mucin/microlumens but no well-formed lumens or glands Moderate cytologic atypia, brisk mitoses, and apoptotic bodies Brisk acute inflammatory infiltrate common Often confused with adenosquamous carcinoma Mixed Not infrequently a mixture of HPVA-related subtypes is seen HPVA, not otherwise specified Does not fit into any other of the above categories but it is confirmed to be HPVA related Note that >1 variant may coexist in a tumor Non–HPV-unassociated (HPV-independent) endocervical adenocarcinoma (HPVI) Gastric-type endocervical adenocarcinoma, ∼10% of all endocervical adenocarcinomas in an international series NO easily identified apical mitotic figures and karyorrhexis at 40× magnification Glands lined by large, columnar cells, frequently containing pink-to-clear mucin, with crisp cytoplasmic borders (plant-like) and atypical nuclei Range of differentiation, from well-formed glands without obvious desmoplasia (“minimal deviation” mucinous adenocarcinoma) to those containing goblet cells and neuroendocrine-type granules, and those showing poor differentiation in the form of fragmented glands and single cells with obvious desmoplasia Confirmation of HPV negativity may be sought, as described above Clear cell carcinoma, ∼3% of all endocervical adenocarcinoma in an international series NO easily identified apical mitotic figures and karyorrhexis at 40× magnification Typical clear cell carcinoma morphology Confirmation of HPV negativity may be sought, as described above Mesonephric carcinoma, < 3% of all endocervical adenocarcinoma, in an international series NO easily identified apical mitotic figures and karyorrhexis at 40X magnification Mixture of patterns (microglandular, glandular, papillary, glomeruloid, spindled), typical of mesonephric carcinoma Confirmation of HPV negativity may be sought, as described above Endometrioid carcinoma, <2% of all endocervical adenocarcinoma in an international series NO easily identified apical mitotic figures and karyorrhexis at 40X magnification Endometrioid-associated features present: endometriosis, low-grade endometrioid glands, squamous differentiation, secretory change, frequently ER/PR positive EXCLUDE extension from lower uterine segment and uterine corpus Confirmation of HPV negativity may be sought, as described above Non-HPVA, not otherwise specified Does not fit into any other of the above categories but it is confirmed to be non-HPV related

### Silva Pattern of Invasion in HPVA Endocervical Adenocarcinoma

This website included a training set of 15 scanned slides and 15 test slides; for the former the diagnosis was provided to participants at the time they viewed the slide whereas for the latter 15 slides no diagnosis was provided; instead participants selected a diagnosis from a drop-down menu and only when the test set was completed would the participant receive their tabulated results, indicating which cases they diagnosed correctly and incorrectly. The training set includes 5 pattern A, 4 pattern B, and 6 pattern C tumors. Upon going to the website, http://www.gpec.ubc.ca/eac, and before viewing the scanned slides, participants received written guidelines on how to classify the pattern of invasion according to the Silva classification system (written by A.R.) (Table 2). For analysis, the participants’ agreement with each of the three patterns was compared (pattern A vs. B vs. C).

**TABLE 2:** Histopathologic assessment of endocervical adenocarcinoma

The purpose of this website is to provide training in performing histopathologic assessment of the pattern of invasion of HPV-associated endocervical adenocarcinoma. This is done by assignment of a pattern based on a 3-tier risk stratification system (*Pathology*. 2018 Feb;50(2):134–140), using the criteria provided below. Pattern A: well-demarcated glands with rounded contours, frequently forming clusters or groups and sometimes showing relatively well-preserved lobular architecture (tumor glands demonstrate a pushing or expansile pattern of invasion). The presence of LVI excludes a tumor from pattern A. Desmoplasia may surround the intact glands in Pattern A. If a tumor is entirely exophytic, with no invasion at the base, it is pattern A. Pattern B: early or limited, localized destructive invasion (individual or small clusters of tumor cells or fragments of glands seen in a desmoplastic, edematous, or inflamed stroma adjacent to an intact gland) Pattern C: diffusely infiltrative glands, with associated extensive, diffuse desmoplastic response (angulated and often incomplete glands open to stroma and/or confluent growth of cribriform or papillary structures within stroma and/or solid or poorly differentiated component)

## RESULTS

### Endocervical Adenocarcinoma: Histopathologic Classification

After construction, the website was tested by 5 expert reviewers who had participated in published studies on the use of IECC. On the basis of this initial expert panel review, 3 cases were excluded/removed from the website for not showing the desired diagnostic features. This resulted in the final website configuration of 25 training slides and 28 test slides.

**FIG. 2:**
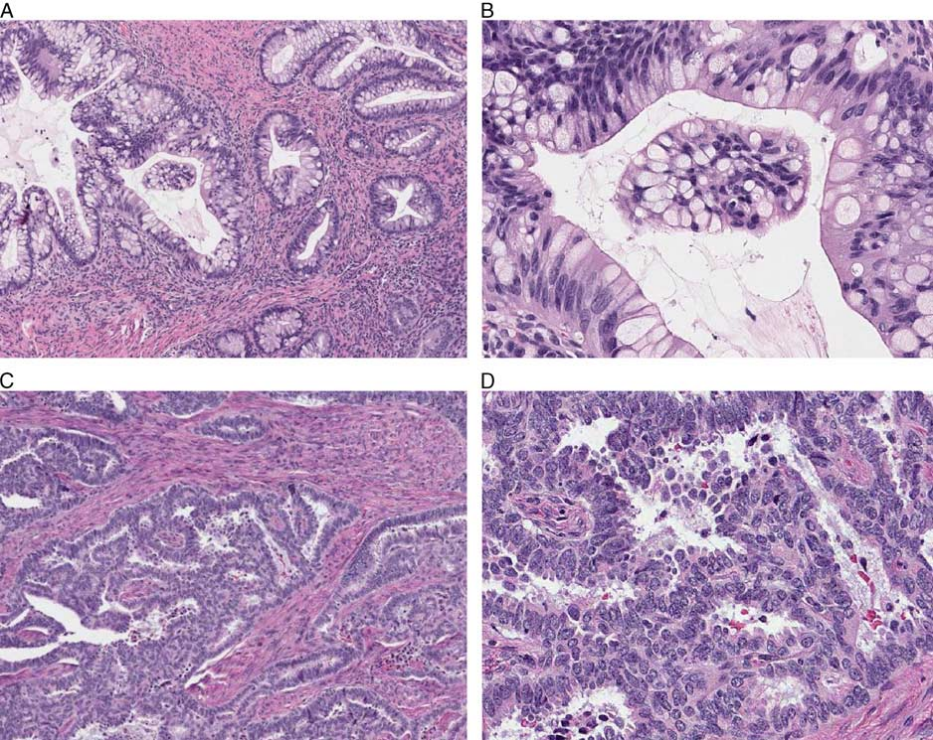
Two cases where there was variability in expert diagnoses of HPV-associated (HPVA) versus HPV-independent (HPVI). (A, B) Case 18 from the test set, diagnosed as HPVA-mucinous (intestinal) by 3 expert reviewers, and HPVI-gastric type by 2. Although there are few mitotic figures present, this tumor was positive for HPV. (C, D) Case 28 from the test set, diagnosed as HPVA-usual type by 2 expert reviewers and HPVI-mesonephric type by 3. This tumor was negative for HPV and showed a mesonephric immunoprofile.

Among the expert reviewers, the diagnostic agreement for HPVA (n=20) versus HPVI (n=8) for the 28 test cases of endocervical adenocarcinoma was near perfect; in 23 cases there was unanimous agreement with respect to HPVA versus HPVI, whereas in 5 cases there were 4 pathologists in agreement and a single dissenting opinion. The accuracy was therefore 96% (135/140 correct diagnoses). With respect to HPVA subtypes (n=20 HPVA cases), there was perfect agreement in 4 cases, 80% agreement in 4 cases, 60% agreement in 8 cases, and 40% agreement in 4 cases (accuracy=68%, 68/100). Considering just the 4 cases of invasive stratified mucinous carcinoma in the study, there was diagnostic agreement in 13/20 (65%). The disagreements included diagnosis of other patterns of HPVA (n=3) and HPVI (n=4, including gastric type (n=3) and mesonephric (n=1)). For the diagnosis of HPVI subtypes (n=8 cases), there was complete agreement among the 5 experts in 4 cases, 80% agreement in 2 cases, and 60% agreement in 2 cases (accuracy=85%). The main diagnostic issues identified were in the diagnosis of HPVI-mesonephric versus HPVI-clear cell, HPVA-mucinous (intestinal) versus HPVI-gastric type, and HPVI-mesonephric versus HPVA (Fig. 2).

**FIG. 3:**
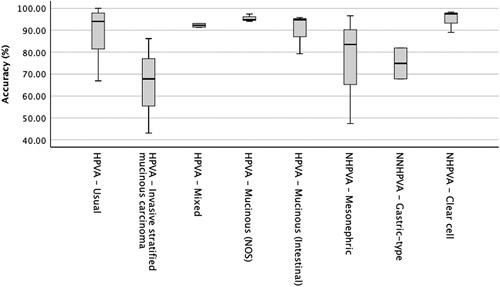
Boxplot of diagnostic accuracy in the subtype diagnosis of endocervical adenocarcinomas. The horizontal line indicates the median, the box is the interquartile values, and the vertical line is the range of results. HPVA indicates HPV-associated; NHPVA, non-HPVA; NOS, not otherwise specified.

There were results for 118 participants in application of the new histopathologic classification criteria through the website. There were 3304 (28 test cases x 118 participants) data points, with missing diagnoses for 30 cases (0.9%). Participants correctly diagnosed HVPA versus HPVI in 86% of cases (87% of HPVA and 83% of HPVI correctly diagnosed). Figure 3 shows a graphical distribution of diagnostic accuracy for the individual categories. The diagnosis of HPVA subtypes was made with 51% accuracy (1132/2218), whereas HPVI subtypes were diagnosed with 62% accuracy (656/1056). A majority of participants agreed with the reference diagnosis in all but two cases; case 40, an example of HPVA-invasive stratified mucinous carcinoma, and case 56, an example of HPVI-mesonephric, which were diagnosed with 43% and 47% accuracy, respectively.

### Silva Pattern of Invasion in HPVA Endocervical Adenocarcinoma

After construction, the website was tested by expert reviewers who had participated in published studies on the diagnosis of Silva invasion patterns. There were 15 training and 15 test cases. The experts showed perfect agreement for 8 of the test cases, 4 of 5 agreed in 4 cases, 3 of 5 experts agreed in 1 case, and there were 2 cases where only 2 of the 5 observers agreed. The diagnostic accuracy was therefore 84% (63/75). The main diagnostic challenge identified was with classification of pattern A versus pattern B. For the clinically relevant classification of pattern A (no significant chance of lymph node metastasis) versus pattern B or C (significant likelihood of lymph node metastasis), there was a consensus in 8/15 cases and 87% diagnostic accuracy (65/75).

**FIG. 4:**
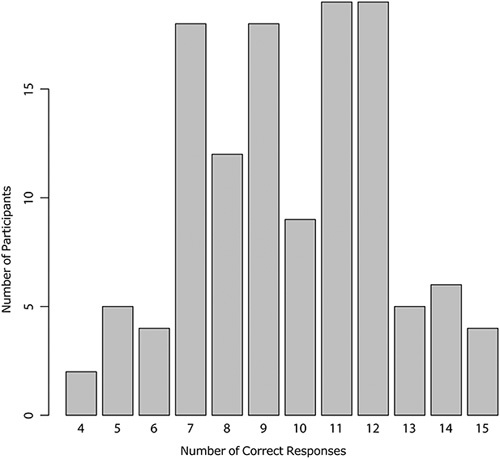
The number of cases (out of 15 in the test set) for which the Silva pattern of invasion was correctly diagnosed is shown for each participant.

**FIG. 5:**
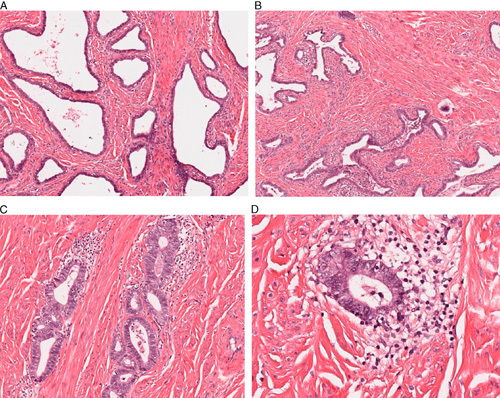
Case 7: This tumor shows predominantly Silva pattern A (A, B). While there is focal stromal response around the invasive glands (C, D), they have smooth contours and this finding is insufficient to warrant diagnosis as pattern B.

**FIG. 6:**
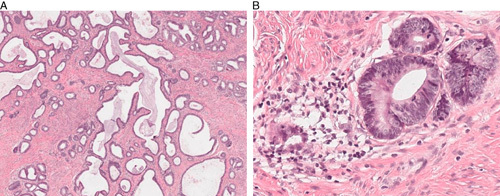
Case 8: This tumor is predominantly Silva pattern A (A), with only focal equivocal evidence of invasion (B), a finding that should not lead to a diagnosis of pattern B.

There were results for 114 participants who applied the Silva classification system to the 15 test cases on the website. There was no missing data. The correct Silva pattern was diagnosed in 67% of cases (1114/1710). The number of correct responses for each participant is shown in Figure 4, with most participants having 7 to 12 of 15 diagnoses correct. There were 4 cases where less than half of the participants had the correct diagnosis (cases 18, 20, 22, and 26, all pattern A). As with the experts, the distinction between pattern A and pattern B was the greatest challenge; for the 6 test cases in which the reference diagnosis was pattern A, the participant’s diagnosis was pattern A in less than half (321/684, 47%) (Figs. 5, 6).

## DISCUSSION

The introduction of international classification systems for gynecologic cancers, allowing for uniform diagnostic criteria and terminology to be used around the world, was identified by Dr Steven Silverberg as one of the most important advances in gynecologic pathology of the past 50 yr 18. Just as standardized staging systems allow for patient outcomes to be compared between different centers, standardized pathologic classification of tumors allows for comparison of cases between centers resulting in more rapid advancement of knowledge. The WHO classification system for tumors is the most widely used system 1. The fifth edition of the WHO Classification of Female Genital Tumours introduces significant changes in the classification of endocervical adenocarcinomas, allowing for more accurate prognostication, which can influence management decisions. The introduction of new classification systems does, however, present a challenge in knowledge translation, that is, how best to promote their rapid uptake and accurate use.

The International Society of Gynecological Pathologists has created 2 sites, available through the society website, that allow for training in these 2 classification systems, using cases diagnosed by experts, followed by self-assessment on an independent set of cases where the correct diagnosis was not provided until the test set had been completed. Besides serving as an educational function, we now present the results of users (initially expert users who were involved in the development of the IECC and Silva classification systems, respectively, and then all those who used the website, irrespective of their level of experience), in an attempt to identify areas that proved particularly diagnostically challenging.

Among expert reviewers, there is excellent agreement in the diagnosis of HPVA versus HPVI endocervical adenocarcinoma. HPVA subtypes were relatively poorly reproducible whereas the accuracy of diagnosis of HPVI subtypes was intermediate. These same trends were observed in more than 100 users of the website. Arguably, the distinction between HPVA and HPVI is the most important one. HPVI adenocarcinomas are much less common than HPVAs. Having recognized an uncommon HPVI adenocarcinoma would allow the observer to seek consultation for a rare tumor type and perform the appropriate confirmatory immunostains. These HPVI adenocarcinomas have probably been underdiagnosed in the past and most have only recently been the subject of extensive research.

The subtypes of HPVA are not associated with differences in prognosis, with the exception of invasive stratified mucinous carcinoma (iSMILE/iSMC), which has been shown to be associated with a worse prognosis 19–21 and those adenocarcinomas showing a mucinous or micropapillary pattern of invasion, which are associated with more or less favorable prognoses, respectively 12. It will be important to ensure that there are robust diagnostic criteria for these entities so that appropriate treatment decisions can be made.

The main challenge in the use of the Silva system of pattern-based assessment of invasion in HPVA adenocarcinoma was distinction between patterns A and B, and this was true both for the experts and the 114 participants. This is the most clinically important distinction, in that it divides HPVA into cases with negligible chance of lymph node metastasis and HPVA where the likelihood of nodal metastasis is such that lymph node dissection is warranted, a critically important therapeutic decision. We observed that there was a tendency to “overdiagnose” pattern B in tumors where the expert opinion was pattern A, and this is clearly an area where further education, for example, a review dedicated just to this distinction, could be impactful. It should be noted that the expert reviewers have noticed a tendency for nonexperts to diagnose focal equivocal destructive invasion as pattern B (Figs. 5, 6), when they would consider such a tumor to be pattern A (unless, of course, there is LVI, in which case the invasion pattern is by definition pattern B). The presence of LVI, while an important prognostic indicator within the Pattern B tumors, was not a focus of this study.

This study has a number of important limitations that should be acknowledged. A single slide per case was available, which may not be as representative. This is particularly important for Silva invasion patterns, as these may vary from slide to slide, and the pattern of invasion for a case is based on the most aggressive pattern identified. Thus, the limited sample for examination, that is a single slide, may have hindered diagnostic performance. The use of whole slide scanned images (scanned at 20×) may have been problematic as pathologists have less experience with digital microscopy than with conventional microscopy. For example, “readily identifiable mitotic figures” is one criterion for the separation of HPVA from HPVI adenocarcinomas, and some observers noted that this was more challenging on whole slide images compared with conventional microscopy, where possible mitoses could more easily be identified by changing the focus slightly (focusing up and down). No attempt was made to select “classic” cases for the website; these were a series of cases from a routine practice setting and included challenging cases. Thus, it can be expected that there are some cases where there will be interobserver variability in opinions, even among experts. There was, however, an effort to ensure that each slide had diagnostic features that would allow for an appropriate diagnostic exercise, as cases with no satisfactory diagnostic features per expert review were removed. Perhaps the most serious limitation, with respect to histopathologic classification, is that participants did not have access to immunohistochemistry results or RNA ISH, which can be invaluable in challenging cases. That said, the IECC system of HPVA versus HPVI endocervical adenocarcinoma and assessment of Silva pattern are both based on examination of hematoxylin & eosin–stained sections. These are intended for use all over the world including health-care systems with limited or no access to ancillary tests. The use of additional tests is discouraged in the IECC in all but the most challenging cases. Likewise, the site was designed to test pathologists individually, without the help from colleagues. Thus, the performance of participants is likely to be less than what would be seen in clinical practice, where access to ancillary testing and consultation with colleagues are available. This study is not a formal test of interobserver reproducibility, which should be done under settings closely mirroring real-life conditions: examining complete slide sets using conventional microscopy, ideally, with the ability to access the types of adjunct testing that is available in routine practice and can be invaluable diagnostically, such as immunohistochemistry.

In conclusion, educational websites developed by the ISGyP, aimed to improve the adoption of relevant classification systems in endocervical adenocarcinoma, have in a short time shown quick interest, with use by more than 100 pathologists so far. On the basis of their results on test sets, there is adequate diagnostic performance overall; however, a number of challenging areas were identified: (1) diagnosis of rare HPVI subtypes, especially mesonephric carcinoma and (2) distinction between Silva pattern A and pattern B. Educational initiatives targeting these areas may result in further improvements in diagnostic performance in the histopathologic assessment of endocervical adenocarcinomas.
